# Fluid biomarkers in cerebral amyloid angiopathy

**DOI:** 10.3389/fnins.2024.1347320

**Published:** 2024-01-26

**Authors:** Seyed Mehrdad Savar, Bin Ma, Eugene Hone, Farzana Jahan, Shaun Markovic, Steve Pedrini, Soudabeh Shemehsavar, Vandhana Easwaran, Kevin Taddei, Samantha Gardener, Jasmeer P. Chhatwal, Ellis S. van Etten, Matthias J. P. van Osch, Daniel Clarke, Anastazija Gnjec, Mark A. van Buchem, Marieke J. H. Wermer, Graeme J. Hankey, Steven M. Greenberg, Ralph N. Martins, Hamid R. Sohrabi

**Affiliations:** ^1^Centre for Healthy Ageing, Health Future Institute, Murdoch University, Perth, WA, Australia; ^2^School of Psychology, Murdoch University, Murdoch, WA, Australia; ^3^School of Medical, Molecular and Forensic Sciences, Murdoch University, Murdoch, WA, Australia; ^4^School of Medical and Health Sciences, Sarich Neuroscience Research Institute, Edith Cowan University, Nedlands, WA, Australia; ^5^College of Science, Technology, Engineering and Mathematics, Murdoch University, Perth, WA, Australia; ^6^Australian Alzheimer's Research Foundation, Nedlands, WA, Australia; ^7^Massachusetts General Hospital, Harvard Medical School, Boston, MA, United States; ^8^Department of Neurology, Leiden University Medical Center, Leiden, Netherlands; ^9^Department of Radiology, Leiden University Medical Center, Leiden, Netherlands; ^10^Sir Charles Gairdner Hospital, Perth Neurology Center, Nedlands, WA, Australia; ^11^Department of Neurology, University Medical Center Groningen, Groningen, Netherlands; ^12^Medical School, The University of Western Australia, Crawley, WA, Australia; ^13^Perron Institute for Neurological and Translational Science, Nedlands, WA, Australia; ^14^Haemorrhagic Stroke Research Program, Massachusetts General Hospital Stroke Research Center, Boston, MA, United States; ^15^Macquarie Medical School, Macquarie University, North Ryde, NSW, Australia

**Keywords:** cerebral amyloid angiopathy, amyloid beta, familial cerebral amyloid angiopathy, differential diagnosis, fluid biomarkers, surrogate endpoints

## Abstract

Cerebral amyloid angiopathy (CAA) is a type of cerebrovascular disorder characterised by the accumulation of amyloid within the leptomeninges and small/medium-sized cerebral blood vessels. Typically, cerebral haemorrhages are one of the first clinical manifestations of CAA, posing a considerable challenge to the timely diagnosis of CAA as the bleedings only occur during the later disease stages. Fluid biomarkers may change prior to imaging biomarkers, and therefore, they could be the future of CAA diagnosis. Additionally, they can be used as primary outcome markers in prospective clinical trials. Among fluid biomarkers, blood-based biomarkers offer a distinct advantage over cerebrospinal fluid biomarkers as they do not require a procedure as invasive as a lumbar puncture. This article aimed to provide an overview of the present clinical data concerning fluid biomarkers associated with CAA and point out the direction of future studies. Among all the biomarkers discussed, amyloid β, neurofilament light chain, matrix metalloproteinases, complement 3, uric acid, and lactadherin demonstrated the most promising evidence. However, the field of fluid biomarkers for CAA is an under-researched area, and in most cases, there are only one or two studies on each of the biomarkers mentioned in this review. Additionally, a small sample size is a common limitation of the discussed studies. Hence, it is hard to reach a solid conclusion on the clinical significance of each biomarker at different stages of the disease or in various subpopulations of CAA. In order to overcome this issue, larger longitudinal and multicentered studies are needed.

## 1 Introduction

### 1.1 Cerebral amyloid angiopathy: pathogenesis, clinical manifestations, and epidemiology

Proteins that are considered amyloidogenic can undergo conformational changes, leading to the formation of β-sheet aggregates. The aggregated amyloidogenic proteins make insoluble fibrils called amyloid fibrils, which are involvedin a number of neurodegenerative diseases, including Alzheimer’s disease (AD), in which amyloid β (Aβ) is the amyloidogenic protein (Chiti and Dobson, 2006; Harrison et al., 2007).

Another condition that is caused by Aβ is cerebral amyloid angiopathy (CAA). CAA is the result of Aβ deposition in leptomeningeal and cortical blood vessel walls, resulting in haemorrhages and cognitive decline. The Aβ deposition usually involves small to medium-sized vessels, with or without the involvement of capillaries, namely CAA type I and CAA type II, respectively (Thal et al., 2002; Gatti et al., 2020; Greenberg et al., 2020). Moderate to severe CAA can be seen in about 6% of cognitively normal older adults and in about 50% of patients with AD (Jakel et al., 2022). According to the Religious Orders Study, the prevalence of CAA pathology in autopsied brains is 84.9%, indicating how commonly CAA occurs (Arvanitakis et al., 2011). While sporadic CAA (sCAA) is the more widespread form of the disease, there are also rare hereditary forms of CAA that typically arise as a consequence of a mutation occurring in the amyloid precursor protein (APP) (Jakel et al., 2022). More recently, two other conditions have been added to the CAA spectrum, namely CAA-related inflammation (CAA-ri) and iatrogenic CAA (i-CAA). CAA-ri is the result of an excessive inflammatory response to vascular Aβ deposition, typically manifesting as a subacute cognitive decline. i-CAA is a condition caused by the prion-like transmission of Aß from cadaveric dural grafts (cadaveric dura), cadaveric human growth hormone, or contaminated surgical instruments (Kaushik et al., 2023; Storti et al., 2023).

In both sCAA and hereditary CAA, the clinical presence of lobar microbleeds and intracerebral haemorrhage (ICH) is noteworthy (Biffi, 2022). More than half the patients with lobar ICH have CAA pathology (Jakel et al., 2022). The global incidence of ICH in 2020 was 3.41 million, with Oceania having the highest age-standardised mortality rate (Tsao et al., 2023). ICH and different types of dementias are among the top 20 causes of years of life lost to premature mortality and death in the United States (Tsao et al., 2023). The lobar microbleeds associated with CAA may have a greater impact on health-related quality of life than deep microbleeds related to hypertension (Tang et al., 2011).

### 1.2 Diagnosis, follow-up, and treatment of cerebral amyloid angiopathy

A definite diagnosis of CAA is only possible through postmortem examination of the brain (Charidimou et al., 2022). There have been some advances in finding neuroimaging biomarkers for diagnosing and tracking CAA, such as using the small vessel disease (SVD) score, which is a score ranging from zero to six and based on the presence of lobar cerebral microbleeds, cortical superficial siderosis (cSS), white matter hyperintensities, and centrum semiovale-enlarged perivascular spaces (CSO-EPVS) in MRI (Charidimou et al., 2016; Valenti et al., 2017). Another instance is using positron emission tomography (PET) agent Pittsburgh compound B (PiB) as a measure of amyloid load to track the disease progression in hereditary CAA (Schultz et al., 2019). In addition to PiB, the clinical application of florbetapir and florbetaben, which are ^18^F-labeled amyloid PET ligands, have also been studied in CAA (Charidimou et al., 2018; Jang et al., 2019). Cerebral haemorrhages and other imaging markers including presence of superficial siderosis, increased perivascular spaces and white matter lesions in a multispot pattern, are employed as diagnostic markers for CAA in the Boston criteria 2.0. These criteria provide the chance to diagnose probable and possible CAA via magnetic resonance imaging (MRI) and clinical data without the need for histopathologic analysis of the brain tissue (Charidimou et al., 2022). The Edinburgh criteria use subarachnoid haemorrhage and finger-like projections observed in computed tomography (CT) and *APOE4* genotype to determine the probability of CAA, which can be used in patients with large bleeds if MRI is not available (Rodrigues et al., 2018; Sembill et al., 2022). That said, the Edinburgh criteria may have low sensitivity in small-sized lobar ICH (van Etten et al., 2020).

As for the management of CAA, currently, there are no treatments available for CAA, except for CAA-ri, in which anti-inflammatory and immunomodulatory medications have shown a reduction in inflammation and a better clinical outcome (Regenhardt et al., 2020).

### 1.3 Aim and search strategy

According to the previous section, the current diagnostic criteria are mainly based on neuroimaging and do not incorporate any fluid biomarkers. However, as seen in AD, cerebrospinal fluid (CSF) biomarkers can be used to detect the disease earlier than PET imaging (Schindler et al., 2018). Furthermore, cerebral haemorrhage is believed to happen at the final stages of the disease (Jakel et al., 2022; Koemans et al., 2023). Therefore, there is a need for biomarkers that reflect the CAA pathology and can be used for diagnostic or prognostic purposes at earlier stages. Studying hereditary CAA provides an opportunity to research CAA before any symptoms. That said, due to the variety of APP mutations and the small number of patients with hereditary CAA, studying these populations has its own limitations (Biffi, 2022).

Another limitation of the current diagnostic criteria could be the availability of resources and expertise, as CAA may be underdiagnosed in rural areas compared to urban areas (Nagaraja and Patel, 2021). Having a proper blood biomarker for CAA could probably be beneficial in that regard since a blood test is relatively cheaper (Padala and Newhouse, 2023).

This article aims to review fluid biomarkers of CAA that have been studied in clinical settings so far and discuss their clinical application and potential for future studies by looking into the body of literature supporting the usage of each biomarker in CAA and, if available, their respective sensitivity and specificity. The search strategy was based on using a combination of the Medical Subject Heading (MeSH) term “Cerebral Amyloid Angiopathy” and other relevant MeSH terms, including “Cerebral Amyloid Angiopathy/blood” and “Cerebral Amyloid Angiopathy/cerebrospinal fluid” in the PubMed database. The next search strategy was to search for “Cerebral Amyloid Angiopathy” and “Biomarker” within the title and abstract of the articles. The output of the two search strategies on August 2, 2023, was 68 and 105 articles, respectively. The references of the obtained results were also screened for relevant articles. A complementary search was implemented on Google Scholar, searching for other potential biomarkers known to the authors to enhance the coverage of the review. Preclinical investigations, conference abstracts, presentations, and research papers that were not in English were excluded. In order to present a clear overview and comparison between the included studies, the sample size, sample characteristics, methods, and main findings were recorded.

## 2 Amyloid beta

The transmembrane protein APP can undergo an amyloidogenic pathway by being metabolised via β-secretase and γ-secretase enzymes into Aβ peptides, which could have 37 to 49 amino acid residues (Chen et al., 2017). Based on the framework proposed by Koemans et al., CAA initially begins with cerebrovascular amyloid deposition (Koemans et al., 2023). Several investigations have explored the viability of Aβ, a crucial component in the definition of CAA, as a potential CSF and plasma biomarker for this condition. Each of the studies has subtle differences from the others, and therefore, a summary of their study design and population is also provided for a better understanding of the role of Aβ as a biomarker for CAA.

A study by Verbeek and colleagues in 2009 with 17 CAA patients (Mean age ± Standard deviation (SD): 62.8 ± 11.9), 72 AD patients (Mean age ± SD: 69.4 ± 8.3), and 58 control subjects (Mean age ± SD: 61.0 ± 8.7) investigated whether CSF Aβ could differentiate between healthy controls and the two clinical groups. This study included the CAA patients according to the original Boston criteria instead of the modified or 2.0 version. The CAA group consisted of both sporadic (15 patients) and hereditary (2 patients) CAA. The results indicated that the CSF levels of Aβ42 were decreased in CAA and AD compared to healthy controls (CAA 42.4% of the controls; AD 51.6% of control values). However, the decrease in CSF Aβ40 was only significant for CAA patients compared to AD and controls. The ratio of Aβ40 to Aβ42 was elevated in AD (182% of control) and CAA (192% of control), but it could not differentiate the two conditions (Verbeek et al., 2009).

A similar study recruited CAA patients using the modified Boston criteria (possible and probable) but on an older cohort (mean age of 78 compared to 62.8) (Renard et al., 2012). CSF Aβ40 and Aβ42 levels in this study were lower in the CAA group compared to the control group. However, the difference was only significant for Aβ42 (Area under the receiver operating characteristic curve (AUC) = 0.75). Similar to the previous research, CSF Aβ40 was significantly lower in CAA patients compared to AD (AUC = 0.795). Additionally, no significant difference was observed between CAA patients with lobar haemorrhage and superficial siderosis with regard to the studied CSF biomarkers (Renard et al., 2012).

A small study with 10 CAA patients (mean age ± SD: 68.6 ± 3), 20 AD patients (mean age ± SD: 62.5 ± 4.1), and ten healthy controls‌ (mean age ± SD: 62.2 ± 5.4) concluded that the median CSF concentrations of Aβ38, Aβ40, and Aβ42 were significantly lower in CAA patients compared to the other two groups. In contrast, AD and the control group had a similar CSF profile regarding the aforementioned biomarkers (Banerjee et al., 2020).

Examining the shared findings among the three investigations mentioned above, it appears that CSF levels of Aβ40 could act as a specific marker for CAA. In fact, this is in line with post-mortem analysis of the human brain tissue, where Aβ40 deposition was more profound in CAA than in AD (Gkanatsiou et al., 2019). That said, those studies did not reject the idea of using Aβ42, which reflects amyloid plaque formation in AD pathology, for differentiating CAA from controls (Gkanatsiou et al., 2019).

One of the largest studies examining the variations in CSF-biomarkers between CAA and AD patients was conducted using a retrospective cohort in two French centres with 63 probable CAA patients (mean age ± SD: 72.6 ± 7.4), 27 AD patients (mean age ± SD: 64.1 ± 6.3), and 21 controls (mean age ± SD: 65.8 ± 9.6). CAA patients recruited in this study had three different CSF profiles. About half the patients (32 patients out of 63) had a CSF profile similar to AD, about a third of them (22 patients out of 63) had isolated decreased Aβ42 levels, and the rest (9 patients out of 63) had normal Aβ42 levels. Aβ40 levels were significantly higher in CAA patients with normal Aβ42 CSF profiles compared to participants with decreased Aβ42 levels. The number of cerebral haemorrhages, including ICH and microbleeds, and the presence of superficial siderosis did not differ between the groups. Additionally, Aβ40 and Aβ42 levels were not correlated with the number of microbleeds detected by MRI. Critically, while Aβ40 levels were not significantly different between AD and controls, they were able to distinguish CAA patients from the other comparison groups, confirming the previous findings (Grangeon et al., 2022a).

In the largest study to date, conducted on 372 participants (67 with CAA, 76 with probable AD, 75 with mild cognitive impairment (MCI) due to AD, 76 with MCI, and 78 healthy controls), Sembill and colleagues reported that CSF levels of both Aβ40 and Aβ42 were lower in CAA compared to healthy controls. However, unlike Aβ40, Aβ42 was comparable in CAA, MCI due to AD, and AD patients. The authors suggested that both Aβ40 and Aβ42 could be used for diagnostic purposes as they could properly distinguish CAA patients from controls (Aβ40: AUC = 0.83, 95%CI (0.76–0.89), *p* < 0.001; Aβ42: AUC = 0.82, 95%CI (0.75–0.88), *p* < 0.001) (Sembill et al., 2023).

Aβ levels have also been investigated in CAA-related inflammation (CAA-ri). It has been reported that Aβ40 levels in the CSF are negatively correlated with amyloid load measured via PET scan when using pons as a reference. There was also a negative, non-significant correlation between CSF Aβ40 levels and the number of microbleeds in MRI (Renard et al., 2018). When comparing CAA-ri to non-inflammatory CAA, CSF Aβ42 levels are lower in CAA-ri (373.3 pg./mL vs. 490.8 pg./mL, *p* = 0.05) (Grangeon et al., 2022b). On the contrary, no significant difference has been reported when comparing CSF Aβ40 and Aβ42 in patients with acute CAA-ri (48 patients with a median age of 77 [71–84] years) and CAA patients with AD (48 patients with a median age of 71 [59–82] years) (Sakai et al., 2023). The similarity of CSF Aβ40 and Aβ42 in CAA-ri and CAA + AD patients suggests that these two biomarkers may not be useful for distinguishing the two populations, yet they can reflect the response to anti-inflammatory treatment in CAA-ri (Sakai et al., 2023).

An important sub-population of hereditary CAA is the Dutch-type CAA (D-CAA). Research has shown that Aβ38, Aβ40, Aβ42, and Aβ43 levels are correlated with each other, and they are lower in both sCAA patients and D-CAA patients compared to controls (De Kort et al., 2023b). However, sCAA and D-CAA patients did not follow the same pattern when it came to SVD burden, as all Aβ levels in sCAA patients were correlated with SVD score, while in D-CAA patients there was only a correlation between Aβ42 and SVD score. Furthermore, except for Aβ43, all the other peptides were significantly lower in sCAA compared to AD (De Kort et al., 2023b).

In a 2017 study, 10 symptomatic D-CAA patients (mean age ± SD: 55 ± 6) were compared to 5 presymptomatic D-CAA patients (mean age ± SD: 36 ± 13) and their age-matched healthy controls regarding their CSF profile. Aβ40 and Aβ42 levels were lower in symptomatic and presymptomatic D-CAA patients compared to their respective controls. Furthermore, presymptomatic individuals reported higher decreases in Aβ42 than Aβ40 at least a decade before the first symptoms of CAA (van Etten et al., 2017). Notably, CSF Aβ40 and Aβ42 levels did not correlate with the number of ICHs quantified via MRI, yet Aβ40 was correlated with the number of microbleeds and white matter hyperintensity volume in an age-adjusted analysis (van Etten et al., 2017).

Bornebroek et al. (2003) compared plasma levels of Aβ40 and Aβ42 in seven asymptomatic D-CAA mutation carriers (mean age: 51 years) to 13 non-carriers (mean age: 57 years). The authors found that Aβ40 was the only amyloid-based biomarker significantly lower in D-CAA plasma samples compared to healthy controls (Bornebroek et al., 2003). In line with this result, it is hypothesised that plasma levels of Aβ40 may initially decrease and then rise in later haemorrhagic stages of CAA (Muir et al., 2023).

Analysing plasma levels of Aβ40 and Aβ42 via the Single Molecule Array (SIMOA) platform in 9 presymptomatic D-CAA mutation carriers (mean age ± SD: 44.11 ± 4.31) and 8 D-CAA mutation non-carriers (mean age ± SD: 43.5 ± 6.57) from the Dominantly Inherited Alzheimer Network (DIAN) study demonstrated promising results using plasma samples, rather than CSF. Both plasma Aβ40 and Aβ42 levels were significantly lower in mutation carriers than controls (Chatterjee et al., 2021b). Similar results were seen in comparing symptomatic and asymptomatic D-CAA patients to controls, where plasma levels of Aβ38, Aβ40, and Aβ42 were all significantly decreased in addition to demonstrating strong inter-correlations suggesting that a panel of these biomarkers could reflect disease progression (Verbeek et al., 2021).

A recent publication with a larger sample size (54 D-CAA patients, 61 sCAA patients) than the aforementioned studies (Bornebroek et al., 2003; Verbeek et al., 2021; Chatterjee et al., 2021b) has provided a more comprehensive understanding of the differences in plasma Aβ expression between hereditary and sporadic CAA. Specifically, plasma levels of Aβ38, Aβ40, and Aβ42 were not significantly lower in presymptomatic D-CAA patients than in age-matched controls. Similar observations were made in the case of sCAA patients and their respective control groups. Notably, a substantial decrease in plasma concentrations of Aβ42 was only evident in symptomatic individuals with D-CAA. Consequently, the outcomes of this investigation propose the potential utility of plasma Aβ42 levels as a promising biomarker for identifying individuals with symptomatic D-CAA (De Kort et al., 2023a). In addition, plasma levels of Aβ in patients with D-CAA were correlated with specific imaging findings such as the Fazekas score and categorised number of lobar cerebral microbleeds but not the microbleed count. Yet, the plasma Aβ levels did not have a correlation with imaging markers in sCAA patients (De Kort et al., 2023a).

Employing a comprehensive panel of both CSF and plasma Aβ might yield a precise distinction in identifying sCAA, D-CAA, AD, and cognitively normal individuals. However, this supposition necessitates further validation through investigations involving larger cohorts. CSF levels of Aβ37, Aβ38, Aβ40, Aβ42, and Aβ43 were lower in CAA patients compared to AD and controls in almost every study. In contrast, measuring Aβ levels in the blood seems to have limited applications, although reduced plasma levels of Aβ42 may be specific to the pathological progression of D-CAA (De Kort et al., 2023a). Furthermore, studies have shown that plasma and CSF levels of Aβ are not correlated in CAA patients (De Kort et al., 2023a). Hence, the dynamics between plasma and CSF Aβ is an area requiring further investigation.

As shown in Table 1, the reported sensitivity and specificity of amyloid and tau as diagnostic biomarkers for CAA varies substantially across different research studies. This may be due to the small sample sizes, the difference in the mean ages of the participants, and the difference in the populations of interest (D-CAA and sCAA).

**Table 1:** Sensitivity and specificity of amyloid β and tau in cerebral amyloid angiopathy studies.

Study	Comparisons	Biomarker	Direction of change	Sensitivity	Specificity	AUC
Verbeek et al. (2009)	CAA (*n* = 17) and Control (*n* = 58)	CSF Aβ40	Lower in CAA	87.5%	47.1%	0.76
		CSF Aβ42	Lower in CAA	86.2%	94.1%	0.96
		CSF t-tau	Higher in CAA	96.5%	52.9%	0.73
		CSF p-tau181	Higher in CAA	77.6%	58.8%	0.67
	CAA (*n* = 17) and AD (*n* = 72)	CSF Aβ40	Lower in CAA	88.2%	59.7%	0.74
		CSF Aβ42	Lower in CAA	58.8%	86.1%	0.68
		CSF t-tau	Lower in CAA	76.5%	76.4%	0.8
		CSF p-tau181	Lower in CAA	88.2%	56.5%	0.79
Grangeon et al. (2022a)	CAA (*n* = 63) and Control (*n* = 21)	CSF Aβ40	Lower in CAA^a^	50.6%	87.4%	0.69
		CSF Aβ42	Lower in CAA^a^	65.5%	92.4%	0.79
		CSF t-tau	Higher in CAA^a^	43.1%	77.1%	0.6
		CSF p-tau181	Higher in CAA^a^	43.5%	76%	0.56
	CAA (*n* = 63) and AD (*n* = 27)	CSF Aβ40	Lower in CAA	59.8%	84.7%	0.72
		CSF Aβ42	Comparable	48.7%	75.8%	0.62
		CSF t-tau	Lower in CAA	53.3%	82.1%	0.67
		CSF p-tau181	Lower in CAA	55.6%	82.5%	0.69
De Kort et al. (2023b)	Presymptomatic D-CAA (*n* = 10) and Control (*n* = 26)	CSF Aβ38	Lower in D-CAA	100%	100%	1
		CSF Aβ40	Lower in D-CAA	100%	100%	1
		CSF Aβ42	Lower in D-CAA	100%	100%	1
		CSF Aβ43	Lower in D-CAA	100%	100%	1
	symptomatic D-CAA (*n* = 12) and Control (*n* = 28)	CSF Aβ38	Lower in D-CAA	100%	100%	1
		CSF Aβ40	Lower in D-CAA	100%	100%	1
		CSF Aβ42	Lower in D-CAA	100%	100%	1
		CSF Aβ43	Lower in D-CAA	100%	100%	1
Noguchi-Shinohara et al. (2017)	AD+CAA-related microbleed (*n* = 34) and AD without microbleeds (*n* = 54)	CSF Aβ40	Lower in CAA- related microbleed	71.4%	92.9%	–
		CSF Aβ42	Lower in CAA- related microbleed	80%	64.8%	–
		CSF t-tau	Lower in CAA- related microbleed	78.6%	60%	–
		CSF p-tau181	Lower in CAA- related microbleed	86.7%	61.1%	–
Sembill et al. (2023)	CAA (*n* = 67) and AD (*n* = 76)	CSF Aβ40	Lower in CAA	–	–	0.76
		CSF Aβ42	Comparable	–	–	0.75
	CAA (*n* = 67) and Control (*n* = 78)	CSF Aβ40	Lower in CAA	–	–	0.96
		CSF Aβ42	Lower in CAA	–	–	0.95
De Kort et al. (2023a)	Presymptomatic D-CAA (*n* = 11) and Control (*n* = 16)	Plasma Aβ38	Lower in D-CAA	–	–	0.87
		Plasma Aβ40	Lower in D-CAA	–	–	0.77
		Plasma Aβ42	Lower in D-CAA	–	–	0.89
	Symptomatic D-CAA (*n* = 24) and Control (*n* = 24)	Plasma Aβ38	Lower in D-CAA	–	–	0.86
		Plasma Aβ40	Lower in D-CAA	–	–	0.73
		Plasma Aβ42	Lower in D-CAA	–	–	0.85

CSF, cerebrospinal fluid; AUC, area under the receiver operating characteristic curve; CAA, cerebral amyloid angiopathy; AD, Alzheimer’s disease; D-CAA, Dutch type cerebral amyloid angiopathy.

a In this study, about half the patients had CSF profiles similar to AD (low Aβ42, high tau levels), a third had isolated decrease in CSF Aβ42, and the rest had normal Aβ42 profiles.

In addition to relying on single biomarkers, a combination of individual biomarkers could also be considered to optimise sensitivity and specificity levels further. For instance, one study proposed an index comprising of Aβ40 and phosphorylated tau (p-tau) 181 (reg = 0.00013164×Aβ40 + 0.0313×p-tau181) that differentiated CAA patients from the control group the best (Sensitivity = 84.6%, specificity = 76.8%, AUC =0.861) (Renard et al., 2012). Finding the optimum tool for each population requires further investigation.

A frequently measured combination of biomarkers is using the Aβ42/40 ratio, as it could reflect brain amyloidosis using amyloid PET scans in cognitively normal individuals (Schindler et al., 2019). This ratio is hypothesised to account for interindividual differences in both overall Aβ production and CSF dynamics in AD (Blennow and Zetterberg, 2018). In a population-based study with 712 participants, a lower plasma Aβ42/Aβ40 ratio was associated with the level of amyloidosis in individuals with cortical microbleeds (McCarter et al., 2022). Yet, this should be considered with caution as a recent publication on a small CAA cohort (*n* = 12) did not show such discrimination using Aβ42/Aβ40 (Jang et al., 2021). As mentioned before, some studies suggested a decrease in both CSF Aβ40 and Aβ42 in CAA, and that may be the reason for these contradicting results for the Aβ42/Aβ40 ratio as a biomarker for CAA.

The most recent meta-analysis available on the significance of Aβ40, Aβ42, total tau (t-tau), and p-tau181 in CAA is a study from early 2022 (Margraf et al., 2022). The results of this meta-analysis on four studies concluded that a CSF ratio of Aβ42/Aβ40 can accurately differentiate CAA and control groups. Still, the study pointed out that the Aβ40, Aβ42, t-tau, and p-tau181 could not tell AD and CAA apart, and other biomarkers should be used in that regard (Margraf et al., 2022). That said, with more recent publications available, the results of an updated meta-analysis may differ from the aforementioned study.

## 3 Tau

Tau protein, along with Aβ42, is one of the core CSF biomarkers for AD (Molinuevo et al., 2018). A conformational change in tau protein could result in the production of highly toxic monomers prone to aggregation, forming oligomers and less toxic neurofibrillary tangles. The toxic tau monomer and oligomers could cause oxidative stress and synaptic dysfunction (Niewiadomska et al., 2021). Furthermore, tau may have a critical role in mediating Aβ-induced neurodegeneration as no signs of neurodegeneration are observed in tau-depleted neurons that are exposed to Aβ (Rapoport et al., 2002). The same observation has been reported in older adults, where an increase in the severity of CAA and burden of amyloid plaques was associated with higher tau burden and cognitive decline (Rabin et al., 2022). Therefore, most of the studies that measured Aβ as a biomarker for CAA also measured the p-tau181 and t-tau levels.

Discrepancies exist in the outcomes concerning the discriminatory capacity of t-tau and p-tau181 in distinguishing between CAA, AD, and healthy control subjects. Some studies have reported that there is a significant difference between the three groups regarding CSF t-tau levels, where AD patients exhibited the highest levels, while healthy individuals demonstrated the lowest t-tau levels (Verbeek et al., 2009; Renard et al., 2012). Other studies report that CSF t-tau levels follow the same order among the three groups, but the difference between CAA patients and the control group is not significant (Banerjee et al., 2020; Grangeon et al., 2022a).

In a study on AD patients with or without microbleeds, it was observed that CSF levels of p-tau181 were markedly diminished in individuals with cortical microbleeds. Notably, all AD patients exhibiting microbleeds in this study were concurrently diagnosed with CAA (Noguchi-Shinohara et al., 2017). Similar to t-tau, some studies have reported a statistically significant difference between CSF p-tau181 levels in CAA patients compared to controls, whereas other investigations have not confirmed such a distinction (Verbeek et al., 2009; Renard et al., 2012).

Looking at the D-CAA population in particular, it has been reported that CSF t-tau and p-tau181 levels are not significantly different between presymptomatic patients and controls. This observation remains consistent when comparing presymptomatic and symptomatic D-CAA participants. Notably, among symptomatic D-CAA patients, solely p-tau181 levels were reduced in comparison to control subjects (van Etten et al., 2017).

To sum up these findings, tau appears to be involved in limited cases of CAA, as it did not show promising results in the presymptomatic D-CAA population. A study has shown that memory impairment in probable CAA could suggest a tau pathology where there is an elevated tau-PET retention (Schoemaker et al., 2021). Hence, measuring the CSF levels of p-tau181 might be most informative in CAA patients with AD pathology. As for t-tau, larger studies are needed to resolve the current discrepancy in the literature.

## 4 Neuroinflammation and oxidative stress biomarkers

### 4.1 Matrix metalloproteinases

A reason for the Aβ deposition (and thus, reduced concentrations of Aβ seen in CSF) may stem from disruptions within enzymatic or non-enzymatic pathways that typically facilitate the clearance of Aβ (Gatti et al., 2020). One group of enzymes responsible for Aβ clearance are the matrix metalloproteinases (MMPs). MMP-2 and (to a lesser extent) MMP-9 have a proteolytic effect on Aβ *in vitro*, producing soluble fragments (Hernandez-Guillamon et al., 2015). In contrast to this function, MMP-2 and MMP-9 can disrupt the tight junctions of the blood–brain barrier (BBB). The immune cells can then enter the brain and produce cytokines that promote MMP-9 expression, leading to more damage to the BBB and cerebral haemorrhage (Gireud-Goss et al., 2021). In fact, MMPs could activate proinflammatory pathways in various neurodegenerative diseases (Brkic et al., 2015). The *in vivo* inhibition of MMP-9 can enhance Aβ clearance across the BBB (Shackleton et al., 2019). A study by Vervuurt and colleagues published in 2023 measured the CSF levels of MMP-2, MMP-9, and MMP-14 in sporadic and D-CAA patients. They also measured the tissue inhibitors of metalloproteinases (TIMP) levels. When comparing sCAA patients (*n* = 28, median age: 72.3 (65.7–77.1) years) with controls (*n* = 40, median age: 64.2 (56.1–69.8) years), the only notable difference was observed for MMP-2/TIMP-2 and MMP-14/TIMP-2 ratios. Furthermore, the symptomatic D-CAA patients (*n* = 12, median age 58.5 (52.8–65.8) years) had lower MMP-14/TIMP-1 and MMP-14/TIMP-2 ratios than the presymptomatic D-CAA patients (*n* = 11, median age: 38.0 (31.0–52.0) years) and controls (*n* = 28, median age: 60.3 (51.6–65.9) years). The symptomatic D-CAA patients also had lower MMP-14 levels than healthy controls (Vervuurt et al., 2023).

The serum levels of MMPs have also been examined in CAA-related ICH. It was observed that the levels of MMP-2 were notably diminished in CAA-related ICH, whereas MMP-9 levels exhibited significant elevation in comparison to the control group (Xia et al., 2021). Moreover, there was an association between serum MMP-3 levels and lobar cerebral microbleed count. Regarding their connection with neuroimaging and cognitive biomarkers, the serum concentrations of MMPs displayed correlations that did not reach statistical significance (Xia et al., 2021). Two studies have shown that MMPs could be a promising biomarker for CAA in CSF and serum. Nevertheless, it is imperative to acknowledge that quantifying the levels of MMPs in CSF or serum is not the same as measuring their enzymatic activity. This common limitation is applicable to both of the aforementioned investigations (Xia et al., 2021; Vervuurt et al., 2023). Additionally, MMPs may not be a specific biomarker for CAA, as they could play a role in other conditions such as AD, Parkinson’s disease (PD), multiple sclerosis, stroke, and meningitis (Rosenberg, 2009). As mentioned before, MMPs have a dual function, where they could be beneficial in Aβ clearance or harmful in promoting neuroinflammation (Rosenberg, 2009). Hence, their temporal changes during the course of the disease need further investigation.

### 4.2 Transforming growth factor-β1

Preclinical evidence suggests that the cytokine transforming growth factor β1 (TGFβ1), which is produced by astrocytes, is involved in vascular deposition of Aβ in CAA (Wyss-Coray et al., 1997). Howe and colleagues have reported that a blood-based panel of MMPs, TGFβs, and a protein associated with vascular basement membrane called fibronectin could accurately differentiate CAA-related ICH from hypertensive ICH (AUC = 0.81) (Howe et al., 2018). However, there is conflicting evidence suggesting that TGF-β1 cannot differentiate subjects with CAA-related cerebral haemorrhage, hypertension-related cerebral haemorrhage, and controls (Greenberg et al., 2000). These contradictions can be explained by looking into the dual function of TGFβ1. According to the literature, TGFβ1 has synaptogenic and neuroprotective effects and reduces parenchymal Aβ deposition, and yet, it may promote vascular amyloid deposition (Diniz et al., 2019; Kapoor and Chinnathambi, 2023). Furthermore, TGFβ1 might not be a proper biomarker at early stages of AD and autosomal dominant AD (Flanders et al., 1995). Therefore, it is conceivable that TGFβ1 might exhibit restricted utility as a biomarker for CAA at different stages of the disease. More investigations are needed to assess the validity of this hypothesis.

### 4.3 Apolipoproteins

Apolipoprotein D (Apo D) is a glycoprotein that assumes a role in numerous neurological disorders characterised by heightened oxidative stress, such as AD, stroke, and PD (Muffat and Walker, 2010). The association of Apo D and CAA pathology has been established. However, the application of Apo D as a biomarker for CAA is not a straightforward matter, as Apo D levels significantly vary between the two genders and different age groups (Kuiperij et al., 2020).

In addition to Apo D, Apo E and Apo J are two other proteins that highlight the involvement of lipid metabolism in CAA. A study of 60 patients with lobar ICH tried to find the connection between imaging biomarkers of CAA with Apo D and Apo E. Lower levels of Apo E and Apo J in low-density lipoproteins (LDL) fractions of the blood samples showed an association with the cSS extent and CSO-EPVS, respectively (Bonaterra-Pastra et al., 2023).

Apo E ε4 is a well-known genetic risk factor for AD and CAA, affecting Aβ conformation and clearance (Holtzman, 2001). Furthermore, the APO E ε2 allele is associated with an increased risk of CAA-related ICH (Camacho et al., 2019). As such, apolipoproteins may be a key component in CAA pathology, and measuring their dynamic changes during the course of the disease is an area worth exploring.

### 4.4 Complement 3

Complement 3 (C3), responsible for inflammation and blood–brain barrier (BBB) disruption, has been investigated in CAA patients. The microglia, activated in response to the accumulation of Aβ, alongside perivascular macrophages, can produce a number of cytokines, including C3. C3b, which is a fragment of C3, then attaches to Aβ and the complement receptor three on the microglia, forming a complex that can migrate to the vessel walls. High levels of C3 in blood samples of CAA patients indicate that this proposed pathway may have a substantial role in CAA pathology (Saito et al., 2022). The C3b can also tag synapses for elimination by microglial phagocytosis, causing neurodegeneration in cases such as AD. There is clinical evidence of C3 being involved in a number of neurological diseases, including AD, traumatic brain injury, Huntington’s disease, and amyotrophic lateral sclerosis (ALS) (Dalakas et al., 2020).

In a retrospective cohort, serum samples of 16 CAA patients with MCI (mean age ± SD: 76.6 ± 4.9 years) were compared to 39 non-CAA patients with MCI (mean age ± SD: 76.1 ± 7.5 years). The outcomes of this investigation have indicated that elevated C3 levels possess the potential to act as an independent predictor for CAA (Saito et al., 2022). That said, a single study is not enough to draw a solid conclusion. Furthermore, the role of C3 as a diagnostic biomarker in cognitively unimpaired CAA patients also remains to be investigated in the future.

### 4.5 Lactadherin

Lactadherin or milk fat globule-EGF factor 8 (MFG-E8) is a glycoprotein involved in various roles, including tissue regeneration, anti-inflammation, and modulation of neurogenesis (Cheyuo et al., 2019). Lactadherin is also associated with CAA Aβ pathology and, therefore, is proposed as a biomarker for CAA. The lactadherin CSF levels are reported to be lower in CAA patients compared to AD patients and healthy controls (Marazuela et al., 2021). Unlike the CSF levels of lactadherin, its serum levels could not differentiate between AD, CAA, and healthy controls (Marazuela et al., 2021). Lactadherin, known for its anti-inflammatory properties within the CNS, looks like a very promising biomarker for CAA as it was correlated with CSF levels of Aβ 40 and Aβ 42 and could differentiate CAA from AD and controls. The exact role of this protein in CAA remains to be clarified through forthcoming investigations (Marazuela et al., 2021).

### 4.6 Uric acid

Uric acid (UA) is another interesting biomarker candidate. UA is involved in anti-oxidative as well as pro-oxidative pathways, depending on its serum level. UA acts as an anti-oxidant at normal levels but shows cytotoxic effects at higher levels (Mijailovic et al., 2022). It is evident, from studying animal models, that a lower level of UA in the blood is associated with higher amyloid deposition in blood vessels and higher Aβ40 expression. Additionally, a higher level of UA reduced the risk of cerebral haemorrhage by preserving the integrity of blood vessels (Wang et al., 2023).

Accordingly, in a study by Hu and colleagues, 82 CAA patients (according to the modified Boston criteria) with ICH (mean age ± SD: 68.4 ± 8.1 years) were compared to 82 healthy controls (mean age ± SD: 68.3 ± 8.6 years) with regard to their serum UA levels. This study demonstrated a pattern for the UA levels, where probable CAA patients (193 ± 57) had a lower UA concentration compared to possible CAA patients (233 ± 78), while the possible CAA patients, in turn, had lower concentrations compared to healthy controls (309 ± 60). The UA levels, however, were not indicative of the region of haemorrhagic lesions (temporooccipital lesions, frontoparietal lesions, multiple lesions) or the severity of neurologic impairment, measured via the National Institute of Health Stroke Scale and the Glasgow Coma Scale (Hu et al., 2014).

### 4.7 Platelet-derived growth factor receptor-Β

Pericytes are vital cells situated between neurons, astrocytes, and endothelial cells that have many roles, including regulation of the BBB integrity, capillary hemodynamic responses, neuroinflammation, and toxic metabolite clearance (Sweeney et al., 2016). One of the surface antigens expressed by pericytes is platelet-derived growth factor receptor-β (PDGFR-β). In neurodegenerative diseases such as AD, CSF levels of PDGFRβ may rise due to the malfunction or destruction of pericytes (Salmina et al., 2019).

Hence, PDGFRβ has also been investigated as a novel CSF biomarker, potentially capable of differentiating CAA, AD and healthy controls. However, the results were not promising for CAA since this biomarker only differentiated controls from amnestic MCI and AD patients with an AD-positive biomarker profile (De Kort et al., 2022).

High levels of CSF PDGFRβ may be associated with BBB disruption and neuroinflammation. However, it could not act as a biomarker for CAA and did not reflect the pathological stages of AD. That said, it is hard to draw a conclusion from this limited data and more studies are needed to find the exact role of PDGFRβ in aging (De Kort et al., 2022; Cicognola et al., 2023).

### 4.8 Neuroleukin

The accumulation of Aβ also promotes the production of reactive oxygen species and neuroinflammation (Carrano et al., 2011). *In vitro* study of human brain pericytes has shown that Aβ exposure can induce the expression of the neuroleukin (NLK) gene (Rensink et al., 2004). CSF levels of NLK could differentiate amnestic MCI and AD from controls. Conversely, this biomarker proved ineffective in distinguishing between individuals with CAA and the control group (De Kort et al., 2021). Although NLK is expected to promote neuroprotective effects in neurodegenerative disease, CSF levels of NLK did not show promising results as a biomarker for diagnosis or disease progression in PD either (Santaella et al., 2020). That said, it is hard to completely disregard NLK as a biomarker for CAA or a specific subpopulation of CAA, given the limited data available to date.

### 4.9 Iron and other metals

An excessive quantity of metals, particularly iron, is associated with the initiation of oxidative stress and the aggregation of Aβ (Carocci et al., 2018). CSF levels of iron, cobalt, ferritin, manganese, and nickel have been explored in CAA. When comparing CSF iron levels, CAA patients had higher levels than AD patients and healthy controls. Ferritin only showed a significant difference in a comparison when the data was not age-adjusted. However, ferritin was significantly correlated with CSF levels of Aβ42. As for the other metals, only CSF levels of nickel were significantly higher in CAA compared to AD patients in an age-adjusted comparison (Banerjee et al., 2022). In a study on patients referred from a memory clinic, no associations were discovered between haemorrhagic markers in MRI and CSF iron levels (Shams et al., 2020).

The aggregation of Aβ is interconnected with the generation of reactive oxygen species and the induction of neuroinflammatory processes (Carrano et al., 2011). Iron can also increase APP expression, leading to more Aβ aggregation and oxidative stress, and the oxidative stress continues the cycle by increasing Aβ aggregation (Uranga and Salvador, 2018). This mechanism, along with the study on metallomics in CAA, suggests that iron may play a role in CAA pathology (Banerjee et al., 2022).

## 5 Neuronal damage biomarkers

In addition to AD-related neurodegeneration in CAA, ischemic or haemorrhagic insults to the brain can also contribute to neuronal damage and brain atrophy (Smith, 2018). The non-haemorrhagic lesions can be observed via neuroimaging about two decades after the initial Aβ deposition within the cerebral vessel walls, which eventually leads to haemorrhagic injuries after another 10 years (Koemans et al., 2023).

Neurofilament light chain (NfL), a specific biomarker for axonal injury, has shown promising results in predicting ICH recurrence in CAA (AUC = 0.84, 95% CI: 0.74–0.93) (Cheng et al., 2020). Serum NfL level is significantly higher in CAA-related ICH compared to controls and is correlated with disease severity (Cheng et al., 2020). The CSF levels of NfL are higher in CAA patients compared to controls, and among those, the amyloid-PET positive CAA patients showed higher levels than the amyloid-PET negative ones (Banerjee et al., 2020). CAA-ri is another condition in which NfL levels in the CSF can be helpful in differentiating between cognitively normal controls and CAA-ri patients (Plotzker et al., 2021). Additionally, the inflammation present in CAA leads to neuronal injury, where NfL could act as a biomarker for it (Banerjee et al., 2020; Cheng et al., 2020; Plotzker et al., 2021).

Serum and CSF levels of NfL are correlated in a variety of neurodegenerative diseases, including Guillain-Barré syndrome (GBS), ALS, and AD, where the CSF levels are about 100 times higher than the serum (Gaiottino et al., 2013). The utilisation of NfL for a differential diagnosis of CAA from the aforementioned diseases appears to exhibit a lack of specificity. That said, the temporal changes in NfL levels could potentially serve as a promising biomarker for tracking disease progression in CAA, something that needs to be explored further.

Glial fibrillary acidic protein (GFAP), an intermediate filament protein produced by astrocytes, faces an increase in production following neuronal damage. In fact, this biomarker is currently used to assess the severity of traumatic brain injuries (TBIs) (Abdelhak et al., 2022). This plasma biomarker is correlated with the number and density of amyloid plaques in AD. However, until now, GFAP plasma levels have failed to reflect vascular deposition of Aβ (Benedet et al., 2022). Nevertheless, the data available on GFAP, especially in CSF, as a biomarker for CAA is limited, making it challenging to derive a definitive conclusion from the current data.

## 6 Other biomarkers

### 6.1 Metabolites

Metabolomics, or the comprehensive evaluation of endogenous metabolites, is based on measuring the gross alterations of biochemical hemostasis within the body fluids, including blood, thereby offering valuable insights into the pathogenesis of various diseases (Serkova et al., 2011).

One of the most extensive studies on blood-based biomarkers for CAA was published in 2021. This study compared 275 metabolites in 9 presymptomatic D-CAA mutation carriers (mean age ± SD: 44.1 ± 4.3 years) to 8 controls (mean age ± SD: 43.5 ± 6.6 years) from the same pedigree. A total number of 22 metabolites from 6 different subgroups, including acylcarnitines, amino acids, biogenic amines, hexoses, phospholipids, and sphingolipids, were found to have different plasma levels between the D-CAA mutation carriers and non-carriers. However, after correction for multiple comparisons, only spermidine levels remained significantly different between the two studied groups (Chatterjee et al., 2021a). A similar study on a larger cohort is recommended to enhance statistical power for finding other relevant metabolites. Spermidine is a metabolite with anti-inflammation and anti-oxidative properties. This polyamine is an autophagy inducer in microglia and astrocytes. Spermidine has been studied in animal models of various brain disorders, including traumatic brain injury, Parkinson’s disease, and memory deficits (Ghosh et al., 2020). In the AD mice model (APP/PS1 mice), Spermidine successfully inhibited the Aβ degradation and the neuroinflammation caused by microglia (Freitag et al., 2022).

### 6.2 Autoantibodies

A dysregulation in B-cell tolerance checkpoints may result in the formation of plasma cells that can enter the brain and produce autoantibodies. These autoantibodies can play a role in neurological and neurodegenerative diseases (Pruss, 2021).

Anti-amyloid β autoantibodies have been studied in CAA-ri as a diagnostic tool. The CSF levels of anti-amyloid β autoantibodies are increased in CAA-ri, and the levels decrease after remission (Piazza et al., 2013; Carmona-Iragui et al., 2016). Studies conducted by other research groups and with larger datasets are needed to validate these findings. The use case of anti-amyloid β autoantibodies as a biomarker for CAA seems to be limited to CAA-ri as the levels come back to the baseline after the acute inflammatory phase. Yet, this biomarker could perform as well as a PiB-PET scan in CAA-ri (Carmona-Iragui et al., 2016). Given the existence of several autoantibodies that are linked with AD, it is conceivable that numerous autoantibodies may also still remain undiscovered within the context of CAA (San Segundo-Acosta et al., 2021; DeMarshall et al., 2022).

### 6.3 MicroRNAs

An additional group of potential biomarkers with considerable promise for investigation in clinical contexts are microRNAs (miRNAs). These miRNAs, which are non-coding RNAs, hold the potential to participate in the pathological mechanisms of both CAA and AD. While miRNAs could be detected in body fluids such as blood, saliva, and CSF, identifying an optimal subset of miRNAs could be challenging due to the existence of over 2,500 identified miRNA variants (Weldon Furr et al., 2019).

To date, two microRNAs, namely miR-582-3p and miR-892b, associated with APP messenger ribonucleic acid (mRNA) translation, have been identified from blood samples of CAA patients (Nicolas et al., 2016).

Using microRNAs as biomarkers for CAA is an under-studied area that holds great potential in discovering novel biomarkers with high specificity for CAA.

### 6.4 Synaptic proteins

As discussed before, the tau protein is responsible for oxidative stress and synaptic dysfunction in AD (Niewiadomska et al., 2021). To assess synaptic dysfunction in CAA, van den Berg and colleagues conducted a study in 2023, where they quantified the concentrations of 15 synaptic proteins within the CSF of patients with CAA, AD, and cognitively un-impaired controls. The levels of all assessed synaptic proteins were found to be comparable between CAA patients and the control group, with the exception of Neuronal Pentraxin 2 (NPTX2). In contrast, AD patients had elevated levels in 12 out of the 15 investigated proteins compared to controls. This significant observation underscores the potential utility of synaptic proteins as biomarkers for distinguishing between AD and CAA (AUC = 0.987) (van den Berg et al., 2023). Furthermore, this data suggests that CAA may not affect synaptic function as much as AD.

## 7 Limitations and future directions

With the advent of new methods such as SIMOA, it is now possible to simultaneously detect multiple biomarkers, from proteins to small molecules, with high sensitivity and specificity (Wang and Walt, 2020). Using a blood-based biomarker instead of a CSF biomarker could markedly benefit patients as it would require a less invasive procedure. However, as seen in the case of lactadherin, a good CSF biomarker does not necessarily translate to a good blood-based biomarker. Similarly, results from a plasma analysis may not be arbitrarily extrapolated to a CSF analysis and making decisions on the viability of using a potential fluid biomarker that is present in both CSF and blood, requires measurements and establishment of firm correlation of such a biomarker in both fluids.

Thus far, some promising biomarkers for AD, like NLK (in CSF), GFAP (in plasma), tau (in CSF), and synaptic proteins (in CSF), have failed to provide convincing results for their utility in distinguishing CAA from cognitively normal controls, highlighting the differing pathology of CAA and AD. That said, confirmatory studies should be considered due to the limited data available on these biomarkers.

Despite this difference, and although AD and CAA are two different diseases with different aetiologies, the two disorders share many similarities (Greenberg et al., 2020). In AD, the disease progression does not occur in a linear manner. Each contributing pathological process, such as neuroinflammation, has a distinct pattern during the course of the disease. Hence, a different panel of biomarkers may become relevant at each stage of AD (Fan et al., 2017). For example, neuroinflammation and microglial activation during prodromal stages of AD may be a neural defence mechanism, given studies have shown that microglial activation is negatively associated with plasma NfL levels, signifying neuronal injury (Parbo et al., 2020). We hypothesise that this may be the case for CAA as well. Some of the discrepancies between the discussed studies may arise from the differences in the mean ages of the participant in these studies, and thus the disease stage of the patients recruited in them. Therefore, longitudinal studies on CAA patients could present a clearer picture of how each biomarker changes during the course of the disease.

Discovering the similarities and differences between D-CAA and sCAA regarding their respective pathological pathway is another crucial step towards finding a reliable biomarker for CAA. The generalisability of findings in presymptomatic D-CAA patients to sCAA patients requires further investigations (Chatterjee et al., 2021b).

Investigating the inflammatory mechanisms inherent to the progression of CAA needs more attention, as there are not enough data available on the interconnection of all the different pathways. Investigations on inflammatory biomarkers could shed light on the pathophysiology of CAA and may have utility in monitoring disease progression, but probably not for diagnostic purposes as they are not specific to CAA. Additionally, apart from the biomarkers discussed in this paper, there remains a lot to be discovered within the field of biomarkers for CAA. Fibrinogen, which is correlated with Aβ levels in AD patients, might be associated with BBB disruption and brain haemorrhage in CAA patients (Fan et al., 2020; Kozberg et al., 2022). Plasma and platelet levels of phosphatidylcholines have preclinical evidence to be used as a diagnostic biomarker for CAA (Foidl et al., 2020). Neutrophil to leukocyte ratio and the systemic immune-inflammation index (SII) could act as prognostic biomarkers in ICH and potentially CAA-related ICH (Liang et al., 2023; Pereira et al., 2023). A number of highly specific proteins, including norrin and collagen alpha-2, have been identified in post-mortem analysis of brain tissue of patients with CAA type I and translating those results into fluid-based measurements in living CAA patients could potentially result in finding a novel and specific biomarker for CAA (Hondius et al., 2018).

A common limitation of biomarker studies on CAA is the small number of enrolled patients. For instance, Banerjee and Werr’ng’s study on the CSF metallomics profile only had 10 CAA patients (Banerjee et al., 2022). This study provided a novel approach to finding a biomarker in CAA, but without better-powered, more comprehensive studies over a longer period of time, no clear conclusions on the validity of metallomics (or other biomarkers of interest) can be drawn. Recruitment of CAA patients from a single center may be feasible for a small study, but a larger study may require a multicentre design (Banerjee and Werring, 2020). A multicentre design brings researchers from different centres and backgrounds together, providing the opportunity to enhance the study design and protocol. Additionally, due to sampling from a more diverse population, the results of a multicentre study are more generalisable to the whole population compared to a single-center study, and smaller effects with enough statistical power can be detected in the large multicentre studies (Chung et al., 2010).

Another factor that needs to be considered concerning the limitations of the studies presented here is the concomitant diseases within the enrolled populations. For instance, in the study on Apo-D, AD patients with concomitant CAA were not identified, and it would be difficult to know if the observed results were related to CAA alone (Kuiperij et al., 2020).

## 8 Conclusion

**Figure 1:**
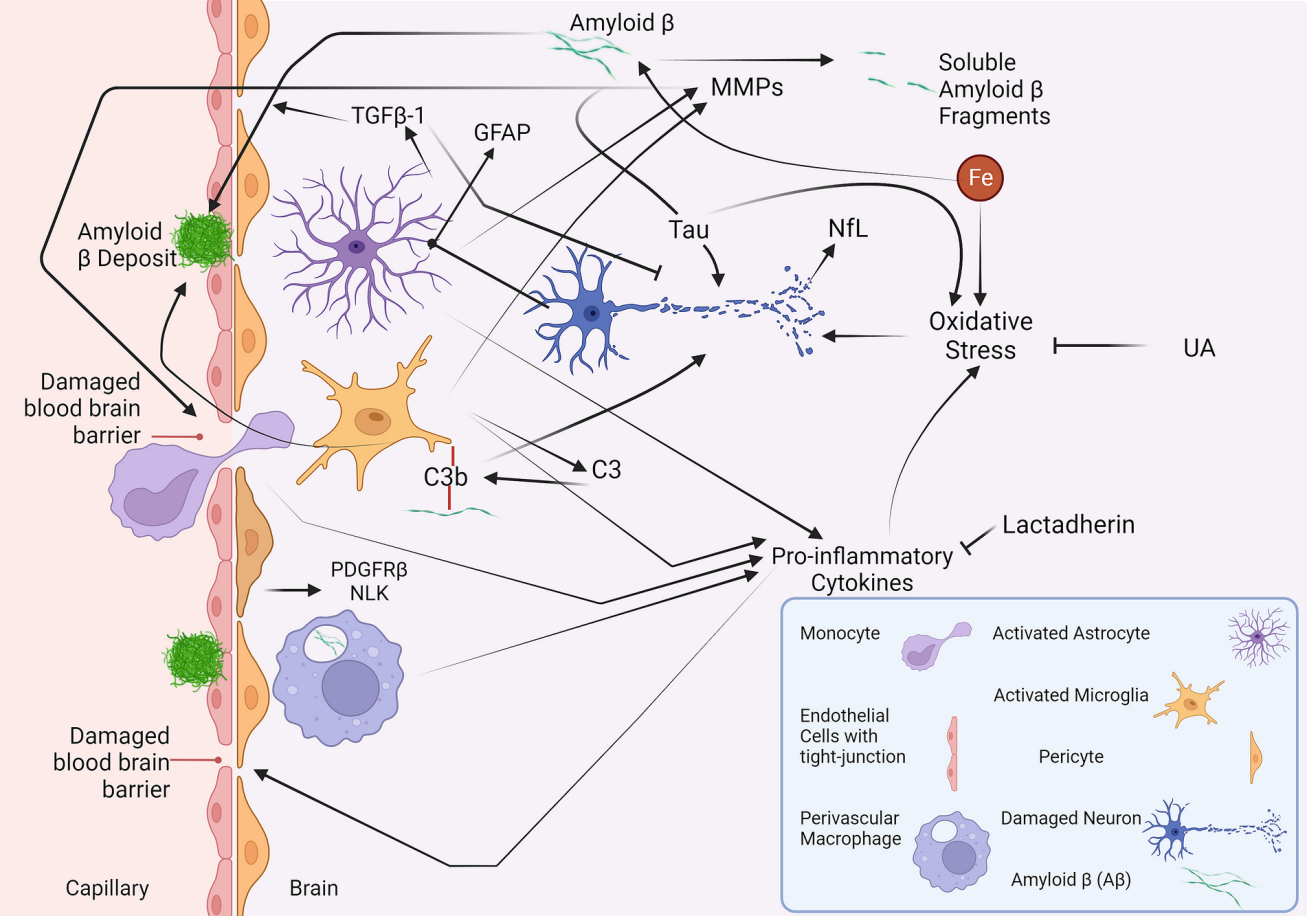
A summary of the fluid biomarkers of cerebral amyloid angiopathy (CAA). The vascular deposition of Aβ leads to CAA (Greenberg et al., 2020). Iron can promote the production of Aβ, whereas MMPs can break down Aβ into soluble fragments (Hernandez-Guillamon et al., 2015; Uranga and Salvador, 2018). The MMPs could also play a harmful role in disrupting the blood–brain barrier (Gireud-Goss et al., 2021). Aβ may also contribute to neurodegeneration with the aid of tau protein (Rapoport et al., 2002). The oxidative stress induced by iron, pro-inflammatory cytokines, and tau results in neuronal damage (Teleanu et al., 2022). In response to this damage, production of GFAP can be induced in activated astrocytes (Abdelhak et al., 2022). NfL is another neuronal damage marker that could be utilised in CAA (Banerjee et al., 2020; Cheng et al., 2020; Plotzker et al., 2021). TGFβ-1, another protein produced by astrocytes, has a dual function and is involved in vascular deposition and clearance of Aβ and, at the same time, has neuroprotective effects (Diniz et al., 2019; Kapoor and Chinnathambi, 2023). C3, produced by the microglia, may play a role in the vascular deposition of Aβ and neurodegeneration (Saito et al., 2022). NLK and PDGFRβ, released by pericytes, are proposed to increase in response to Aβ and damage to pericytes, respectively (De Kort et al., 2021, 2022). Yet, neither NLK nor PDGFRβ has shown significant changes in CAA. Lactadherin and UA have anti-inflammatory and anti-oxidative features, respectively (Cheyuo et al., 2019; Mijailovic et al., 2022). However, their levels are decreased in CAA (Hu et al., 2014; Marazuela et al., 2021), preventing them from mitigating the neuroinflammation and oxidative stress present in CAA. Aβ, amyloid β; C3, complement 3; GFAP, glial fibrillary acidic protein; MMP, matrix metalloproteinase; NFL, neurofilament light chain; NLK, neuroleukin; PDGFRβ, platelet-derived growth factor receptor-β; TGFβ, transforming growth factor; UA, uric acid.

This review has conducted a comprehensive analysis of the major fluid biomarkers that have been investigated in relation to all subtypes of CAA in a clinical setting (Table 2). Some of the biomarkers discussed in this review and their interactions are illustrated in Figure 1, providing an overview of the ongoing pathophysiological pathways identified in CAA.

**Table 2:** A summary of fluid biomarkers of cerebral amyloid angiopathy.

Biomarker	Mechanism	Association with cerebral amyloid angiopathy	Studies
Amyloid β (Aβ)	Involved in the Pathophysiology of cerebral amyloid angiopathy (CAA)	Aβ is strongly associated with cerebral amyloid angiopathy. Cerebrospinal fluid (CSF) levels of Aβ37, Aβ38, Aβ40, Aβ42, and Aβ43 were lower in CAA patients compared to Alzheimer’s disease (AD) patients and controls in almost every study. Reduced plasma levels of Aβ42 may be specific to D-CAA. The significance of different Aβ species at each stage of CAA may vary.	(Bornebroek et al., 2003; Verbeek et al., 2009; Renard et al., 2012; van Etten et al., 2017; Renard et al., 2018; Gkanatsiou et al., 2019; Banerjee et al., 2020; Jang et al., 2021; Verbeek et al., 2021; Chatterjee et al., 2021b; Margraf et al., 2022; Grangeon et al., 2022a,b; Sakai et al., 2023; Sembill et al., 2023; De Kort et al., 2023a,b)
Tau	May increase amyloid burden and cause oxidative stress	Tau may be involved in limited cases of CAA that have AD pathology. Reports from other cases are inconclusive.	(Verbeek et al., 2009; Renard et al., 2012; Noguchi-Shinohara et al., 2017; van Etten et al., 2017; Banerjee et al., 2020; Schoemaker et al., 2021; Rabin et al., 2022; Grangeon et al., 2022a)
Matrix metalloproteinases (MMPs)	Neuroinflammation, blood–brain barrier disruption, and Aβ clearance	MMPs may play a role in sCAA, D-CAA, and CAA-related intracerebral haemorrhage (ICH) but are not specific to CAA pathology.	(Xia et al., 2021; Vervuurt et al., 2023)
Transforming growth factor β1 (TGFβ1)	Vascular deposition of Amyloid β and neuroprotection	There are contradicting reports on using TGFβ1 as a biomarker in CAA-related ICH.	(Greenberg et al., 2000; Howe et al., 2018)
Apolipoproteins	Oxidative stress	Apolipoproteins are associated with CAA pathology and imaging biomarkers such as cortical superficial siderosis and centrum semiovale-enlarged perivascular spaces in CAA-related lobar ICH. However, they may not be specific to CAA.	(Kuiperij et al., 2020; Bonaterra-Pastra et al., 2023)
Complement 3 (C3)	Neuroinflammation and blood–brain barrier disruption	C3 levels are elevated in CAA patients with mild cognitive impairment (MCI) compared to non-CAA MCI (AUC = 0.68, 95%CI (0.53–0.83)). Yet, C3 is not specific to CAA, and there is not enough data on its role in cognitively unimpaired CAA patients.	(Saito et al., 2022)
Lactadherin	Anti-inflammation	Lactadherin levels in CSF are correlated with Aβ 40 and Aβ 42 and could differentiate CAA, AD, and controls.	(Marazuela et al., 2021)
uric acid	Anti-inflammation	Uric acid could differentiate probable CAA, possible CAA, and healthy controls in CAA-related ICH. However, it was not correlated with the level of neurologic impairment.	(Hu et al., 2014)
Platelet-derived growth factor receptor-β (PDGFR-β)	Neuroinflammation and blood–brain barrier disruption	The current limited data on PDGFR-β does not show an association with CAA pathology.	(De Kort et al., 2022)
Neuroleukin (NLK)	Anti-inflammation and anti-oxidative stress	The current limited data on NLK does not show an association with CAA pathology.	(De Kort et al., 2021)
Iron	May increase amyloid precursor protein expression and oxidative stress	Iron may be involved in CAA pathology, but further investigations are needed.	(Banerjee et al., 2022)
Neurofilament light chain (NfL)	Axonal injury	NfL has shown promising results in CAA, CAA-related ICH and CAA-related inflammation and could reflect disease progression. However, NfL is not a specific biomarker for CAA.	(Banerjee et al., 2020; Cheng et al., 2020; Plotzker et al., 2021)
Glial fibrillary acidic protein (GFAP)	Neuronal damage	Plasma levels of GFAP are not associated with CAA pathology. CSF levels of GFAP need further investigation.	(Benedet et al., 2022)
Spermidine	Anti-inflammation and anti-oxidative	This metabolite has shown promising results in differentiating D-CAA mutation carriers and non-carriers. However, spermidine may not be a specific biomarker for CAA.	(Chatterjee et al., 2021a)
Anti-amyloid β autoantibodies	Neuroinflammation	Anti-Aβ autoantibodies may play a role in CAA-related inflammation.	(Piazza et al., 2013; Carmona-Iragui et al., 2016)
MicroRNAs	Associated with amyloid precursor protein mRNA translation	miR-582-3p and miR-892b are two specific microRNAs observed in blood samples of CAA patients.	(Nicolas et al., 2016)
Synaptic proteins	Synaptic dysfunction	Most of the studied synaptic proteins remain unchanged in CAA, except for NPTX2. A combination of synaptic proteins could differentiate CAA and AD (AUC = 0.987, 95%CI (0.97–1.00))	(van den Berg et al., 2023)

Many biomarkers (e.g., Aβ, UA, C3, MMPs, lactadherin, and NfL) have shown promising evidence in their ability to diagnose and provide a prognosis of disease progression. However, biomarkers that were related to pericytes (NLK and PDGFRβ) did not show promising results, and the results for tau and TGFβ were inconclusive. Additionally, some biomarkers, such as GFAP, autoantibodies, microRNAs, and metabolites, are under-researched and require more investigation. In fact, the current body of evidence for biomarkers of CAA needs an enhancement in both quality and quantity by conducting confirmatory and exploratory studies. Finding a suitable CSF biomarker for CAA reduces the need for special equipment for the diagnosis and follow-up of the patients. A blood-based biomarker has an additional advantage since it requires a less invasive procedure than a lumbar puncture.

Given the differences and similarities between sporadic CAA, hereditary CAA, and AD, it seems that a panel of fluid biomarkers is needed for a differential diagnosis instead of a single fluid biomarker. Additionally, these biomarkers could add to the accuracy of existing diagnostic criteria that are mostly based on imaging biomarkers. Further investigations are needed to identify the optimal combination of biomarkers with high sensitivity and specificity at each stage of the disease. Identifying more sensitive biomarker panels with better clinical utility will help us better characterise CAA and, thus, may help inform future therapeutic avenues. In addition, a reliable biomarker that could reflect the disease progression may be utilised to monitor treatment response effectively and thus help validate new treatment approaches.

## Author contributions

SMS: Conceptualization, Writing – original draft, Writing – review & editing. BM: Conceptualization, Supervision, Writing – review & editing, Project administration. EH: Supervision, Writing – review & editing, Conceptualization. FJ: Writing – review & editing, Supervision. SM: Writing – review & editing. SP: Writing – review & editing. SoS: Writing – review & editing. VE: Writing – review & editing. KT: Writing – review & editing. SaG: Writing – review & editing. JC: Writing – review & editing. EE: Writing – review & editing. MO: Writing – review & editing. DC: Writing – review & editing. AG: Writing – review & editing. MB: Writing – review & editing. MW: Writing – review & editing. GH: Writing – review & editing. StG: Conceptualization, Writing – review & editing. RM: Conceptualization, Supervision, Writing – review & editing. HS: Conceptualization, Project administration, Supervision, Writing – review & editing.
